# Tetra­kis(μ-pivalato-κ^2^ *O*:*O*′)bis­[(2-methyl­pyridine-κ*N*)iron(II)](*Fe*—*Fe*)

**DOI:** 10.1107/S1600536808005102

**Published:** 2008-02-27

**Authors:** Jacob Overgaard, Grigore A. Timco, Finn K. Larsen

**Affiliations:** a Department of Chemistry, University of Aarhus, Langelandsgade 140, DK-8000 Aarhus C, Denmark; b Institute of Chemistry, Academy of Sciences of Moldova, Academy Str. 3, Chisinau, MD-2028, Republic of Moldova

## Abstract

The asymmetric unit of the title compound, [Fe_2_(C_5_H_9_O_2_)_4_(C_6_H_7_N)_2_], contains one unique Fe-atom site located close to a centre of symmetry which generates the mol­ecular dimer. The two Fe atoms are bridged by four carboxyl­ate groups and are each coordinated by a mol­ecule of 2-picoline. Electron counting and the 18-electron rule suggest that a chemical single bond is likely to exist between the two Fe atoms, which are separated by a distance of 2.8576 (4) Å. This bond completes an approximately octa­hedral coordination environment around each Fe atom.

## Related literature

For related literature, see: Celengil-Cetin *et al.* (2000 ▶); Weber (1980 ▶); Johnson (1976 ▶). 
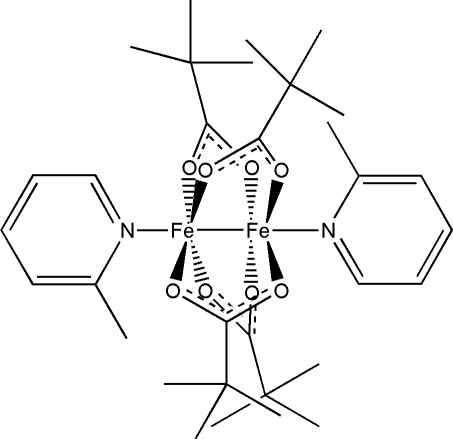


## Experimental

### 

#### Crystal data

[Fe_2_(C_5_H_9_O_2_)_4_(C_6_H_7_N)_2_]
*M* *_r_* = 702.44
Triclinic, 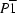

*a* = 9.5387 (8) Å
*b* = 10.5403 (9) Å
*c* = 10.5546 (9) Å
α = 64.138 (2)°
β = 83.600 (2)°
γ = 72.090 (2)°
*V* = 908.30 (13) Å^3^
*Z* = 1
Mo *K*α radiation
μ = 0.84 mm^−1^
*T* = 120 (2) K
0.30 × 0.25 × 0.20 mm

#### Data collection

Bruker SMART 1000 CCD area-detector diffractometer
Absorption correction: Gaussian (*SADABS*; Sheldrick, 2003 ▶) *T*_min_ = 0.761, *T*_max_ = 0.863
8222 measured reflections
4949 independent reflections
4259 reflections with *I* > 2σ(*I*)
*R*_int_ = 0.032

#### Refinement

*R*[*F* ^2^ > 2σ(*F* ^2^)] = 0.035
*wR*(*F* ^2^) = 0.101
*S* = 1.10
4949 reflections
206 parameters
H-atom parameters constrained
Δρ_max_ = 0.56 e Å^−3^
Δρ_min_ = −0.48 e Å^−3^

### 

Data collection: *SMART* (Bruker, 1998 ▶); cell refinement: *SAINT-Plus* (Bruker, 1998 ▶); data reduction: *SAINT-Plus*; program(s) used to solve structure: *SHELXS97* (Sheldrick, 2008 ▶); program(s) used to refine structure: *SHELXL97* (Sheldrick, 2008 ▶); molecular graphics: *XP* in *SHELXTL* (Sheldrick, 2008 ▶); software used to prepare material for publication: *SHELXL97*.

## Supplementary Material

Crystal structure: contains datablocks global, I. DOI: 10.1107/S1600536808005102/wk2077sup1.cif

Structure factors: contains datablocks I. DOI: 10.1107/S1600536808005102/wk2077Isup2.hkl

Additional supplementary materials:  crystallographic information; 3D view; checkCIF report

## Figures and Tables

**Table 1:** Selected bond lengths (Å)

Fe1—O4^i^	2.0508 (11)
Fe1—O1	2.0571 (13)
Fe1—O3	2.0675 (13)
Fe1—O2^i^	2.0717 (11)
Fe1—N1	2.1284 (13)
Fe1—Fe1^i^	2.8576 (4)

Symmetry code: (i) 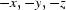.

**Fig. 1.**
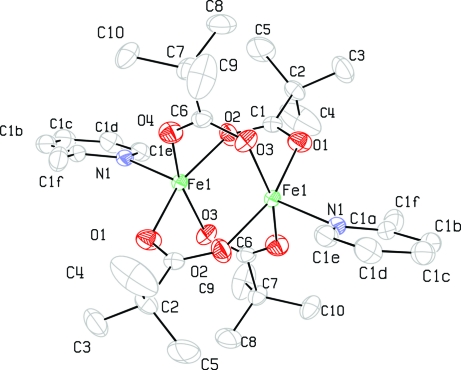
ORTEPdrawing (Johnson, 1976) of (1) showing the atomic labelling scheme. Hydrogen atoms are omitted for clarity. The thermal ellipsoids show 50% probability surfaces.

## supplementary crystallographic information

### Comment

In a systematic study of the reactions of iron powder with simple carboxylates and aromatic amines to prepare iron(II) carboxylates, the title compound, (1) (Fig. 1) resulted, under ambient reaction conditions. Both monomeric molecular complexes (Celengil-Cetin *et al.*, 2000) and extended iron(II) carboxylates have previously been prepared (Weber, 1980) using similar methods.

In the present study, the iron atoms are only coordinated to five ligands each, with a total donation of 10 electrons. This gives a total of 16 electrons on both Fe, thus a Fe—Fe bond needs to be present to fill the outer orbitals on Fe. The relatively short Fe(1)—Fe(1) interaction distance (*d*(Fe(1)–Fe(1)) = 2.8576 (4) Å) is likely evidence for such iron-iron bond.

### Experimental

Iron powder (1.0 g) was refluxed in a solution of 2-picoline (C_6_H_7_N, 10.0 ml), pivalic acid (4.0 g) and water (1.0 ml) for 5 h under an inert atmosphere. The obtained yellow solution was filtered while hot under an inert atmosphere and the filtrate afforded green crystals upon cooling slowly at room temperature.

### Refinement

The methyl hydrogen atoms were constrained to tetrahedral geometry with C—H distances of 0.98 Å and *U*_iso_(H) = 1.5*U*_eq_(C). The positions of each set of three of the H atoms of the methyl groups constrained to tetrahedral geometry were refined so as to optimize the overlap with the observed electron density (AFIX 137). The H atoms bonded to the aromatic C atoms were constrained to ride on their parent atom in a distance of 0.95 Å in an ideal geometry and with *U*_iso_(H) = 1.2 *U*_eq_(C).

### Crystal data

**Table:**

[Fe_2_(C_5_H_9_O_2_)_4_(C_6_H_7_N)_2_]	*Z* = 1
*M**_r_* = 702.44	*F*_000_ = 372
Triclinic, *P*1	*D*_x_ = 1.284 Mg m^−^^3^
*a* = 9.5387 (8) Å	Mo *K*α radiation λ = 0.71073 Å
*b* = 10.5403 (9) Å	Cell parameters from 2580 reflections
*c* = 10.5546 (9) Å	θ = 2.9–32.7º
α = 64.138 (2)º	µ = 0.85 mm^−^^1^
β = 83.600 (2)º	*T* = 120 (2) K
γ = 72.090 (2)º	Prism, green
*V* = 908.30 (13) Å^3^	0.30 × 0.25 × 0.20 mm

### Data collection

**Table:**

Bruker SMART 1000 CCD area-detector diffractometer	4949 independent reflections
Radiation source: fine-focus sealed tube	4259 reflections with *I* > 2σ(*I*)
Monochromator: graphite	*R*_int_ = 0.032
Detector resolution: 12.0 pixels mm^-1^	θ_max_ = 33.8º
*T* = 120(2) K	θ_min_ = 2.2º
ω scans	*h* = −11→12
Absorption correction: gaussian(SADABS; Sheldrick, 2003)	*k* = −13→16
*T*_min_ = 0.762, *T*_max_ = 0.863	*l* = −14→12
8222 measured reflections	

### Refinement

**Table:**

Refinement on *F*^2^	Secondary atom site location: difference Fourier map
Least-squares matrix: full	Hydrogen site location: inferred from neighbouring sites
*R*[*F*^2^ > 2σ(*F*^2^)] = 0.035	H-atom parameters constrained
*wR*(*F*^2^) = 0.101	*w* = 1/[σ^2^(*F*_o_^2^) + (0.0621*P*)^2^ + 0.0412*P*] where *P* = (*F*_o_^2^ + 2*F*_c_^2^)/3
*S* = 1.11	(Δ/σ)_max_ < 0.001
4949 reflections	Δρ_max_ = 0.56 e Å^−^^3^
206 parameters	Δρ_min_ = −0.48 e Å^−^^3^
Primary atom site location: structure-invariant direct methods	Extinction correction: none

### Special details

**Table:**

Geometry. All e.s.d.'s (except the e.s.d. in the dihedral angle between two l.s. planes) are estimated using the full covariance matrix. The cell e.s.d.'s are taken into account individually in the estimation of e.s.d.'s in distances, angles and torsion angles; correlations between e.s.d.'s in cell parameters are only used when they are defined by crystal symmetry. An approximate (isotropic) treatment of cell e.s.d.'s is used for estimating e.s.d.'s involving l.s. planes.
Refinement. Refinement of *F*^2^ against ALL reflections. The weighted *R*-factor *wR* and goodness of fit *S* are based on *F*^2^, conventional *R*-factors *R* are based on *F*, with *F* set to zero for negative *F*^2^. The threshold expression of *F*^2^ > σ(*F*^2^) is used only for calculating *R*-factors(gt) *etc*. and is not relevant to the choice of reflections for refinement. *R*-factors based on *F*^2^ are statistically about twice as large as those based on *F*, and *R*- factors based on ALL data will be even larger.

### Fractional atomic coordinates and isotropic or equivalent isotropic displacement parameters (Å^2^)

**Table:**

	*x*	*y*	*z*	*U*_iso_*/*U*_eq_	
Fe1	−0.03369 (2)	−0.11318 (2)	−0.02000 (2)	0.02183 (8)	
O1	0.16294 (13)	−0.13551 (14)	−0.12201 (14)	0.0380 (3)	
O2	0.20582 (14)	0.04754 (14)	−0.09692 (13)	0.0355 (3)	
O3	0.08500 (15)	−0.25206 (16)	0.16687 (14)	0.0443 (3)	
O4	0.12814 (14)	−0.07109 (13)	0.19692 (12)	0.0360 (3)	
C1	0.23705 (16)	−0.04856 (16)	−0.14308 (15)	0.0246 (3)	
C2	0.37218 (19)	−0.05517 (19)	−0.23535 (17)	0.0327 (3)	
C3	0.4232 (3)	−0.2015 (3)	−0.2491 (3)	0.0555 (6)	
H3A	0.3466	−0.2079	−0.2987	0.083*	
H3B	0.4415	−0.2836	−0.1551	0.083*	
H3C	0.5142	−0.2064	−0.3023	0.083*	
C4	0.3273 (4)	0.0754 (3)	−0.3758 (2)	0.1009 (14)	
H4A	0.2995	0.1665	−0.3629	0.151*	
H4B	0.2432	0.0693	−0.4161	0.151*	
H4C	0.4101	0.0753	−0.4397	0.151*	
C5	0.4987 (2)	−0.0447 (3)	−0.1668 (3)	0.0671 (7)	
H5A	0.4659	0.0437	−0.1491	0.101*	
H5B	0.5823	−0.0389	−0.23	0.101*	
H5C	0.5288	−0.1321	−0.0775	0.101*	
C6	0.14310 (16)	−0.20469 (18)	0.23159 (15)	0.0271 (3)	
C7	0.24543 (19)	−0.31837 (17)	0.35736 (15)	0.0303 (3)	
C8	0.3883 (2)	−0.3772 (3)	0.2947 (2)	0.0602 (6)	
H8A	0.4266	−0.2953	0.2316	0.09*	
H8B	0.3695	−0.427	0.2415	0.09*	
H8C	0.461	−0.4473	0.3706	0.09*	
C9	0.1777 (4)	−0.4420 (3)	0.4476 (2)	0.0679 (8)	
H9A	0.2495	−0.5209	0.5203	0.102*	
H9B	0.1501	−0.481	0.3881	0.102*	
H9C	0.0897	−0.4036	0.4924	0.102*	
C10	0.2741 (2)	−0.2478 (2)	0.44773 (18)	0.0409 (4)	
H10A	0.3193	−0.1694	0.3904	0.061*	
H10B	0.3407	−0.3227	0.527	0.061*	
H10C	0.1806	−0.2062	0.4837	0.061*	
N1	−0.12992 (14)	−0.26829 (14)	−0.02513 (14)	0.0262 (3)	
C1A	−0.12609 (18)	−0.30575 (18)	−0.13207 (18)	0.0300 (3)	
C1B	−0.1759 (2)	−0.4233 (2)	−0.1168 (2)	0.0386 (4)	
H1BA	−0.171	−0.4493	−0.1932	0.046*	
C1C	−0.2317 (2)	−0.5006 (2)	0.0088 (2)	0.0424 (4)	
H1CA	−0.2643	−0.5815	0.0207	0.051*	
C1D	−0.2402 (2)	−0.4603 (2)	0.1183 (2)	0.0414 (4)	
H1DA	−0.2815	−0.5108	0.2053	0.05*	
C1E	−0.18751 (19)	−0.34521 (18)	0.09860 (19)	0.0336 (3)	
H1EA	−0.1915	−0.3185	0.1743	0.04*	
C1F	−0.0673 (2)	−0.2167 (2)	−0.26681 (18)	0.0397 (4)	
H1FA	0.0329	−0.2192	−0.2505	0.06*	
H1FB	−0.1305	−0.1146	−0.304	0.06*	
H1FC	−0.0653	−0.2573	−0.3349	0.06*	

### Atomic displacement parameters (Å^2^)

**Table:**

	*U*^11^	*U*^22^	*U*^33^	*U*^12^	*U*^13^	*U*^23^
Fe1	0.01980 (12)	0.02218 (11)	0.02716 (12)	−0.00602 (8)	−0.00176 (8)	−0.01322 (8)
O1	0.0264 (6)	0.0324 (6)	0.0575 (8)	−0.0128 (5)	0.0049 (5)	−0.0193 (6)
O2	0.0342 (7)	0.0406 (7)	0.0408 (6)	−0.0122 (5)	0.0116 (5)	−0.0268 (5)
O3	0.0387 (7)	0.0543 (8)	0.0456 (7)	−0.0036 (6)	−0.0154 (6)	−0.0291 (6)
O4	0.0388 (7)	0.0291 (6)	0.0288 (5)	−0.0013 (5)	−0.0068 (5)	−0.0061 (4)
C1	0.0199 (7)	0.0263 (7)	0.0239 (6)	−0.0055 (5)	0.0008 (5)	−0.0081 (5)
C2	0.0295 (8)	0.0347 (8)	0.0314 (8)	−0.0075 (7)	0.0112 (6)	−0.0154 (6)
C3	0.0481 (12)	0.0612 (14)	0.0708 (14)	−0.0102 (11)	0.0179 (11)	−0.0477 (12)
C4	0.102 (2)	0.085 (2)	0.0341 (11)	0.0216 (17)	0.0300 (13)	0.0076 (12)
C5	0.0283 (10)	0.0836 (18)	0.110 (2)	−0.0247 (11)	0.0200 (12)	−0.0580 (17)
C6	0.0203 (7)	0.0364 (8)	0.0215 (6)	−0.0050 (6)	0.0007 (5)	−0.0116 (6)
C7	0.0361 (9)	0.0281 (7)	0.0224 (6)	−0.0077 (6)	−0.0070 (6)	−0.0061 (5)
C8	0.0391 (11)	0.0744 (16)	0.0519 (12)	0.0178 (11)	−0.0188 (9)	−0.0318 (11)
C9	0.125 (2)	0.0509 (13)	0.0361 (10)	−0.0530 (15)	0.0006 (12)	−0.0069 (9)
C10	0.0517 (11)	0.0413 (9)	0.0287 (8)	−0.0139 (8)	−0.0127 (7)	−0.0108 (7)
N1	0.0215 (6)	0.0253 (6)	0.0350 (7)	−0.0048 (5)	−0.0023 (5)	−0.0163 (5)
C1A	0.0244 (7)	0.0309 (7)	0.0388 (8)	−0.0025 (6)	−0.0056 (6)	−0.0206 (6)
C1B	0.0306 (9)	0.0334 (8)	0.0588 (11)	−0.0045 (7)	−0.0072 (8)	−0.0270 (8)
C1C	0.0331 (9)	0.0320 (8)	0.0685 (13)	−0.0079 (7)	−0.0033 (9)	−0.0269 (9)
C1D	0.0367 (10)	0.0317 (8)	0.0540 (11)	−0.0134 (7)	0.0088 (8)	−0.0158 (8)
C1E	0.0286 (8)	0.0300 (8)	0.0428 (9)	−0.0083 (7)	0.0031 (7)	−0.0167 (7)
C1F	0.0470 (11)	0.0437 (10)	0.0353 (8)	−0.0130 (8)	−0.0012 (8)	−0.0224 (8)

### Geometric parameters (Å, °)

**Table:**

Fe1—O4^i^	2.0508 (11)	C7—C8	1.522 (3)
Fe1—O1	2.0571 (13)	C7—C9	1.523 (3)
Fe1—O3	2.0675 (13)	C7—C10	1.529 (2)
Fe1—O2^i^	2.0717 (11)	C8—H8A	0.98
Fe1—N1	2.1284 (13)	C8—H8B	0.98
Fe1—Fe1^i^	2.8576 (4)	C8—H8C	0.98
O1—C1	1.2548 (19)	C9—H9A	0.98
O2—C1	1.2508 (19)	C9—H9B	0.98
O2—Fe1^i^	2.0717 (11)	C9—H9C	0.98
O3—C6	1.255 (2)	C10—H10A	0.98
O4—C6	1.255 (2)	C10—H10B	0.98
O4—Fe1^i^	2.0508 (11)	C10—H10C	0.98
C1—C2	1.529 (2)	N1—C1A	1.342 (2)
C2—C4	1.508 (3)	N1—C1E	1.362 (2)
C2—C5	1.529 (3)	C1A—C1B	1.399 (2)
C2—C3	1.535 (3)	C1A—C1F	1.483 (2)
C3—H3A	0.98	C1B—C1C	1.368 (3)
C3—H3B	0.98	C1B—H1BA	0.95
C3—H3C	0.98	C1C—C1D	1.382 (3)
C4—H4A	0.98	C1C—H1CA	0.95
C4—H4B	0.98	C1D—C1E	1.378 (2)
C4—H4C	0.98	C1D—H1DA	0.95
C5—H5A	0.98	C1E—H1EA	0.95
C5—H5B	0.98	C1F—H1FA	0.98
C5—H5C	0.98	C1F—H1FB	0.98
C6—C7	1.527 (2)	C1F—H1FC	0.98
			
O4^i^—Fe1—O1	89.37 (5)	C8—C7—C9	110.6 (2)
O4^i^—Fe1—O3	162.10 (6)	C8—C7—C6	105.56 (13)
O1—Fe1—O3	87.58 (6)	C9—C7—C6	110.04 (16)
O4^i^—Fe1—O2^i^	89.38 (5)	C8—C7—C10	110.31 (17)
O1—Fe1—O2^i^	161.85 (5)	C9—C7—C10	109.23 (15)
O3—Fe1—O2^i^	88.07 (5)	C6—C7—C10	111.10 (14)
O4^i^—Fe1—N1	101.37 (5)	C7—C8—H8A	109.5
O1—Fe1—N1	107.04 (5)	C7—C8—H8B	109.5
O3—Fe1—N1	96.39 (5)	H8A—C8—H8B	109.5
O2^i^—Fe1—N1	90.95 (5)	C7—C8—H8C	109.5
O4^i^—Fe1—Fe1^i^	77.91 (4)	H8A—C8—H8C	109.5
O1—Fe1—Fe1^i^	86.43 (4)	H8B—C8—H8C	109.5
O3—Fe1—Fe1^i^	84.30 (4)	C7—C9—H9A	109.5
O2^i^—Fe1—Fe1^i^	75.61 (4)	C7—C9—H9B	109.5
N1—Fe1—Fe1^i^	166.53 (4)	H9A—C9—H9B	109.5
C1—O1—Fe1	119.93 (11)	C7—C9—H9C	109.5
C1—O2—Fe1^i^	133.33 (11)	H9A—C9—H9C	109.5
C6—O3—Fe1	121.86 (12)	H9B—C9—H9C	109.5
C6—O4—Fe1^i^	130.76 (11)	C7—C10—H10A	109.5
O2—C1—O1	124.45 (15)	C7—C10—H10B	109.5
O2—C1—C2	116.85 (14)	H10A—C10—H10B	109.5
O1—C1—C2	118.67 (15)	C7—C10—H10C	109.5
C4—C2—C5	110.6 (2)	H10A—C10—H10C	109.5
C4—C2—C1	106.23 (16)	H10B—C10—H10C	109.5
C5—C2—C1	109.10 (15)	C1A—N1—C1E	118.33 (14)
C4—C2—C3	112.1 (2)	C1A—N1—Fe1	126.41 (11)
C5—C2—C3	107.82 (18)	C1E—N1—Fe1	114.92 (11)
C1—C2—C3	111.00 (15)	N1—C1A—C1B	121.33 (16)
C2—C3—H3A	109.5	N1—C1A—C1F	116.98 (15)
C2—C3—H3B	109.5	C1B—C1A—C1F	121.69 (16)
H3A—C3—H3B	109.5	C1C—C1B—C1A	119.52 (17)
C2—C3—H3C	109.5	C1C—C1B—H1BA	120.2
H3A—C3—H3C	109.5	C1A—C1B—H1BA	120.2
H3B—C3—H3C	109.5	C1B—C1C—C1D	119.66 (17)
C2—C4—H4A	109.5	C1B—C1C—H1CA	120.2
C2—C4—H4B	109.5	C1D—C1C—H1CA	120.2
H4A—C4—H4B	109.5	C1E—C1D—C1C	118.50 (17)
C2—C4—H4C	109.5	C1E—C1D—H1DA	120.8
H4A—C4—H4C	109.5	C1C—C1D—H1DA	120.8
H4B—C4—H4C	109.5	N1—C1E—C1D	122.61 (17)
C2—C5—H5A	109.5	N1—C1E—H1EA	118.7
C2—C5—H5B	109.5	C1D—C1E—H1EA	118.7
H5A—C5—H5B	109.5	C1A—C1F—H1FA	109.5
C2—C5—H5C	109.5	C1A—C1F—H1FB	109.5
H5A—C5—H5C	109.5	H1FA—C1F—H1FB	109.5
H5B—C5—H5C	109.5	C1A—C1F—H1FC	109.5
O3—C6—O4	124.77 (15)	H1FA—C1F—H1FC	109.5
O3—C6—C7	117.45 (15)	H1FB—C1F—H1FC	109.5
O4—C6—C7	117.70 (14)		
			
O4^i^—Fe1—O1—C1	72.98 (13)	O3—C6—C7—C9	−43.7 (2)
O3—Fe1—O1—C1	−89.39 (13)	O4—C6—C7—C9	139.25 (18)
O2^i^—Fe1—O1—C1	−13.1 (2)	O3—C6—C7—C10	−164.77 (15)
N1—Fe1—O1—C1	174.67 (12)	O4—C6—C7—C10	18.2 (2)
Fe1^i^—Fe1—O1—C1	−4.95 (12)	O4^i^—Fe1—N1—C1A	55.44 (13)
O4^i^—Fe1—O3—C6	5.7 (3)	O1—Fe1—N1—C1A	−37.42 (14)
O1—Fe1—O3—C6	86.11 (14)	O3—Fe1—N1—C1A	−126.84 (13)
O2^i^—Fe1—O3—C6	−76.26 (14)	O2^i^—Fe1—N1—C1A	145.00 (13)
N1—Fe1—O3—C6	−167.00 (14)	Fe1^i^—Fe1—N1—C1A	140.97 (14)
Fe1^i^—Fe1—O3—C6	−0.53 (13)	O4^i^—Fe1—N1—C1E	−131.33 (11)
Fe1^i^—O2—C1—O1	−1.9 (3)	O1—Fe1—N1—C1E	135.81 (11)
Fe1^i^—O2—C1—C2	175.95 (10)	O3—Fe1—N1—C1E	46.39 (12)
Fe1—O1—C1—O2	5.5 (2)	O2^i^—Fe1—N1—C1E	−41.77 (12)
Fe1—O1—C1—C2	−172.25 (10)	Fe1^i^—Fe1—N1—C1E	−45.8 (2)
O2—C1—C2—C4	−74.1 (2)	C1E—N1—C1A—C1B	−1.8 (2)
O1—C1—C2—C4	103.8 (2)	Fe1—N1—C1A—C1B	171.27 (12)
O2—C1—C2—C5	45.1 (2)	C1E—N1—C1A—C1F	178.04 (15)
O1—C1—C2—C5	−136.93 (18)	Fe1—N1—C1A—C1F	−8.9 (2)
O2—C1—C2—C3	163.78 (17)	N1—C1A—C1B—C1C	0.9 (3)
O1—C1—C2—C3	−18.3 (2)	C1F—C1A—C1B—C1C	−178.84 (17)
Fe1—O3—C6—O4	5.0 (2)	C1A—C1B—C1C—C1D	1.0 (3)
Fe1—O3—C6—C7	−171.88 (11)	C1B—C1C—C1D—C1E	−2.0 (3)
Fe1^i^—O4—C6—O3	−8.7 (3)	C1A—N1—C1E—C1D	0.7 (2)
Fe1^i^—O4—C6—C7	168.15 (11)	Fe1—N1—C1E—C1D	−173.13 (14)
O3—C6—C7—C8	75.6 (2)	C1C—C1D—C1E—N1	1.2 (3)
O4—C6—C7—C8	−101.42 (19)		

Symmetry codes: (i) −*x*, −*y*, −*z*.
